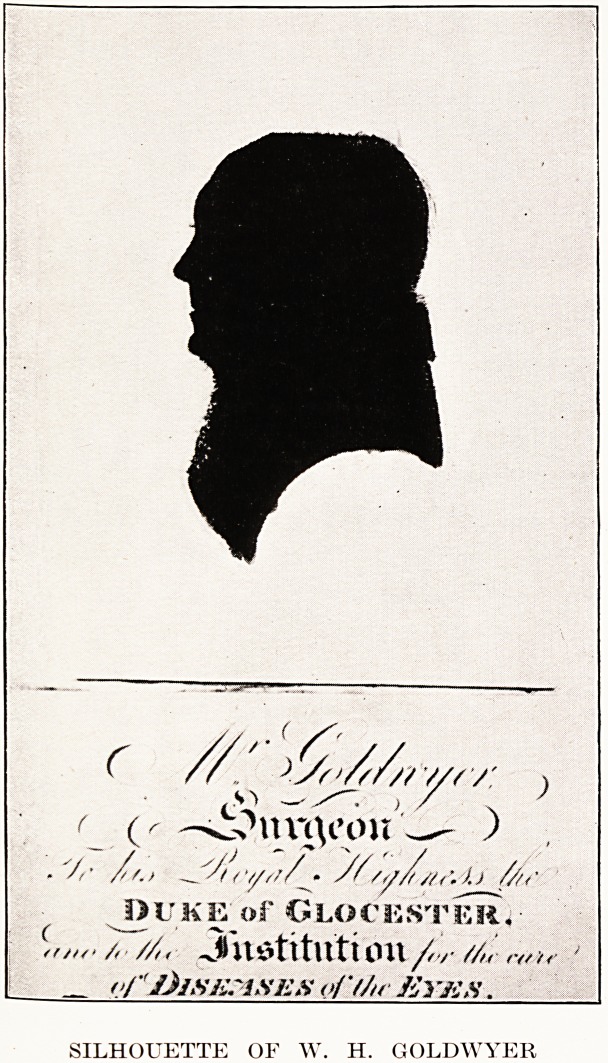# Bristol Eye Hospital
*The speech of the Chairman, Mr. Mortimer Harley, at the opening of the new Hospital by the Duchess of Beaufort, on 21st October, 1935, forms the basis of this article.


**Published:** 1936

**Authors:** 


BRISTOL EYE HOSPITAL.

### BRISTOL EYE HOSPITAL.*

BY

THE EDITOR.

---

THE Bristol Eye Hospital was founded in the year 1810 as the outcome of a public meeting called to determine the propriety of establishing an "Institution for the cure of Diseases of the Eye among the Poor" in Bristol, there being at that time only two similar hospitals in the country, namely the Royal London Ophthalmic Hospital, Moorfields, founded in 1804, and the West of England Eye Infirmary, Exeter, in 1808.

The prime mover in its establishment was William Henry Goldwyer, a member of a family which sent fifteen of its sons in five generations into the medical profession. Goldwyer was a good all round surgeon, engaged like other higher class surgeons of his day in general practice, but in addition he was an excellent accoucheur and a skilful operator on cataracts. In personal appearance he was short, thick-set and round-shouldered, walking with a decided stoop; his eyes were globular and prominent. Acting with great energy, he enlisted the sympathies of many eminent citizens in the project of founding an eye hospital. On the 18th of June, 1810, a meeting was held at the Guildhall; the Duke of Gloucester (George III's nephew) was nominated Patron, Stephen Cave the

* The speech of the Chairman, Mr. Mortimer Harley, at the opening of the new Hospital by the Duchess of Beaufort, on 21st October, 1935, forms the basis of this article.

Treasurer, and Goldwyer the Surgeon-Oculist. Mr. J. M. Tandy was the Secretary. The Hospital was an immediate success. By the end of the year more than two thousand patients had been seen. Goldwyer's services were so universally appreciated that the Freedom of the City was presented to him in 1816. In 1817 the Duchess of Gloucester became Patroness, and was succeeded by the Duke of Beaufort in 1858.

The work of the Hospital, which was started in a house in Lower Maudlin Street, adjoining the Blind Asylum, continued practically unaltered and with very little change for the next seventy-five years. The Out-patient Department consisted of two small rooms on the ground floor, one being the patients' waiting-room, and the other the surgeons' consulting room, whilst above there were only two small rooms, one of which was a small ward for four or five in-patients, and the other was used as the residence of the matron-housekeeper.

Towards the end of the nineteenth century the Eye Hospital had lapsed into a somewhat torpid state. It was re-vitalized by Richardson Cross, who had come to Bristol in 1878 at the invitation of University College to be Lecturer in Anatomy. He was also appointed to the surgical staff of the Royal Infirmary. Soon after his arrival in Bristol he turned his attention to Ophthalmology, and in 1882 was elected Surgeon to the Eye Hospital. Here his ability and driving power quickly made themselves felt. Cross was essentially a man who got things done, because he knew what he wanted, and had a way of inspiring others with faith in his plans. For forty-three years he continued to take an active interest in the affairs of the Hospital, "the institution which," as his biographer in this *Journal* wrote at his death in

SILHOUETTE OF W. H. GOLDWYER

1931, "he rescued from obscurity and to which he devoted so large a portion of his life."

We give here a list of Surgeons who preceded Mr. Cross, with their dates of appointment:—

William Henry Goldwyer, 1810.
Henry Goldwyer, jun., 1816.
William Goldwyer, 1820.
Ralph Bernard, 1845.
Robert T. H. Bartley, 1855.
R. H. Dew, 1869.
F. Richardson Cross, 1882.

One of the first results of Cross's energy was that in 1886 another public meeting was called, it being then evident that the building was inadequate for the number of patients making application for treatment. Support was obtained, and funds, including several anonymous contributions, were raised from numerous sources.

At that time the Committee had considered the building of an entirely new Hospital, but the suggestion was abandoned for fear of leaving the Institution with an overwhelming debt for many years to come. The adjoining house, which was formerly occupied by the Blind Asylum, was purchased and altered, so as to provide wards containing sixteen beds, but the upper part was let.

In 1889 further extensions became necessary, and the second enlargement of the Hospital was made. The upper part of the house was taken in hand and modified. This enlargement was formally opened in April, 1893, by Her Grace the then Duchess of Beaufort. The first House Surgeon, Herman Snellen, son of Professor Snellen of "Test Type" fame, was appointed in 1889. He afterwards succeeded his father as Professor of Ophthalmology in the University of Utrecht.

In 1900 an entirely new story was placed on the building, together with a new Operating Theatre, alterations and extensions being also made in the side facing the garden. This was opened in March, 1901, by the seventh Duke of Beaufort. A few years later the Committee again felt the need of an up-to-date Eye Hospital, but it was not until a sum of £25,000 had been set aside that plans were prepared for a building worthy of the City of Bristol.

The foundation-stone of the new Hospital was laid by Mrs. Edmund King on 27th November, 1933, and now, after two years, there stands in place of a number of dilapidated cottages a modern Eye Hospital worthy of the cause for which the Hospital was founded a century and a quarter ago, "The Cure and Treatment of Diseases of the Eye amongst the Poor of Bristol and District." On 21st October, 1935, as recorded in our Winter issue, 1935, the new Hospital was formally opened by Her Grace the Duchess of Beaufort, at a meeting presided over by the Right Hon. the Lord Mayor of Bristol.

By the courtesy of the Chairman and Committee of the Eye Hospital we are publishing an illustration of the new building, which shows the front of the new Hospital, and on the right of the picture the two houses which formed the Hospital until a year ago, and which have been reconditioned to become the administrative block and nurses' home. By permission of Messrs. Arrowsmith we reproduce the silhouette portrait of Goldwyer from Munro Smith's *History of the Bristol Royal Infirmary.*

## Figures and Tables

**Figure f1:**
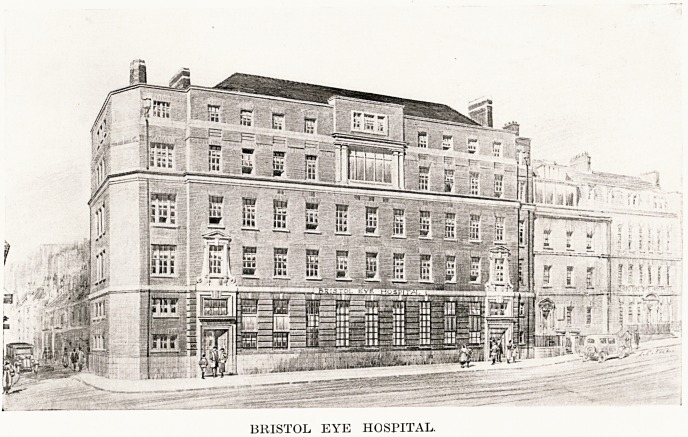


**Figure f2:**